# Supplementary figures and images for: Perception—Action dissociations depend on factors that affect multisensory processing
Source: PLoS One. 2024 Nov 4;19(11):e0301737. doi: 10.1371/journal.pone.0301737 (PMC11534235; doi:10.1371/journal.pone.0301737)

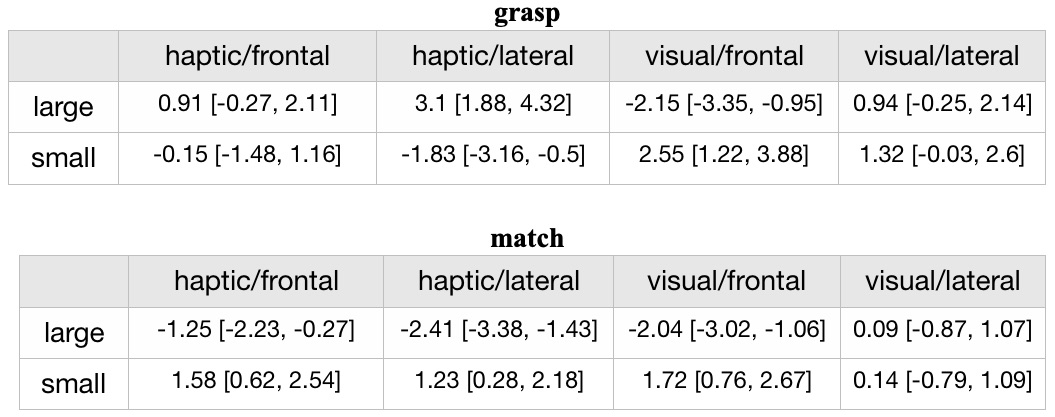

Supplement: S1 Table — Each cell reports exact values of the estimated effect and their confidence intervals (95% CIs) presented in Fig 5. For details see the section 3.1 in the main text. (TIF) [file pone.0301737.s001.tif]

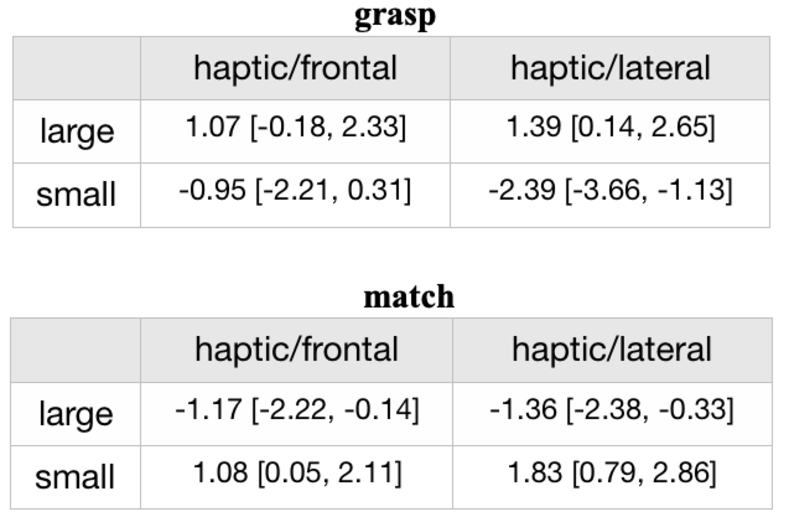

Supplement: S2 Table — Each cell reports exact values of the estimated effect and their confidence intervals (95% CIs) presented in Fig 6. For details see the section 3.2 in the main text. (TIF) [file pone.0301737.s002.tif]

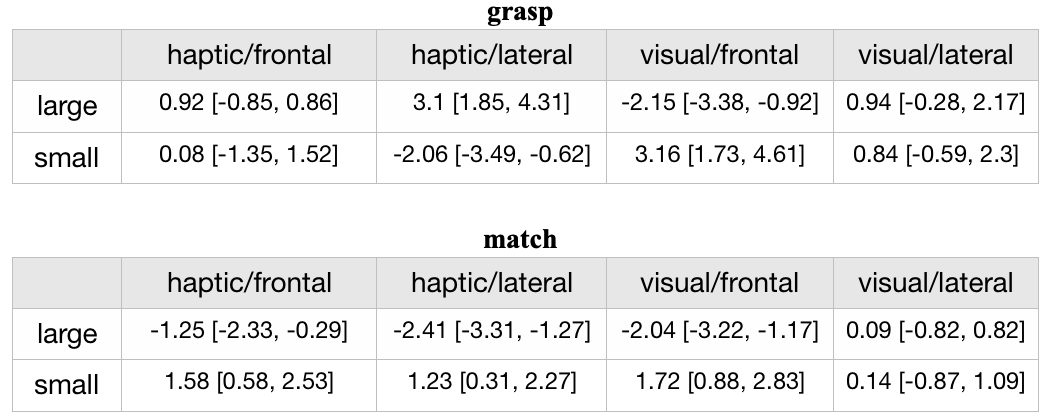

Supplement: S3 Table — Each cell reports exact values of the estimated effect and their confidence intervals (95% CIs) presented in Fig 5. For details see the section 3.1 in the main text. Compare these estimates with those in S1 Table. (TIF) [file pone.0301737.s003.tif]

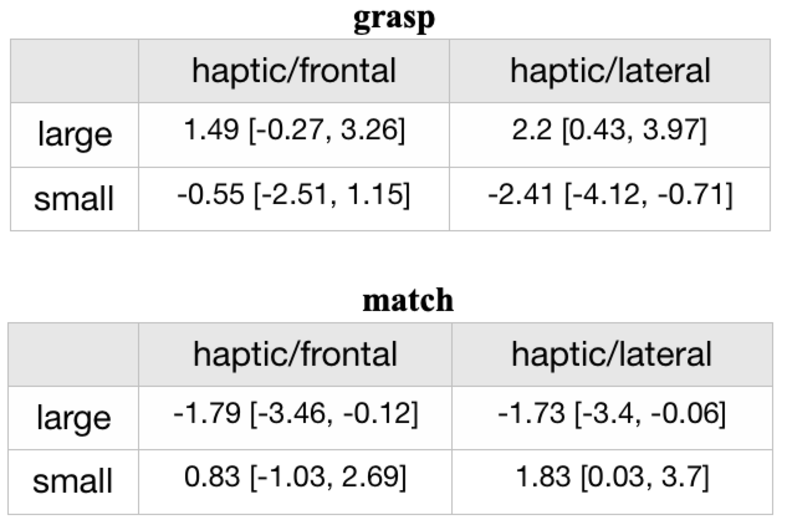

Supplement: S4 Table — Each cell reports exact values of the estimated effect and their confidence intervals (95% CIs) presented in Fig 6. For details see the section 3.2 in the main text. Compare these estimates with those in S2 Table. (TIF) [file pone.0301737.s004.tif]

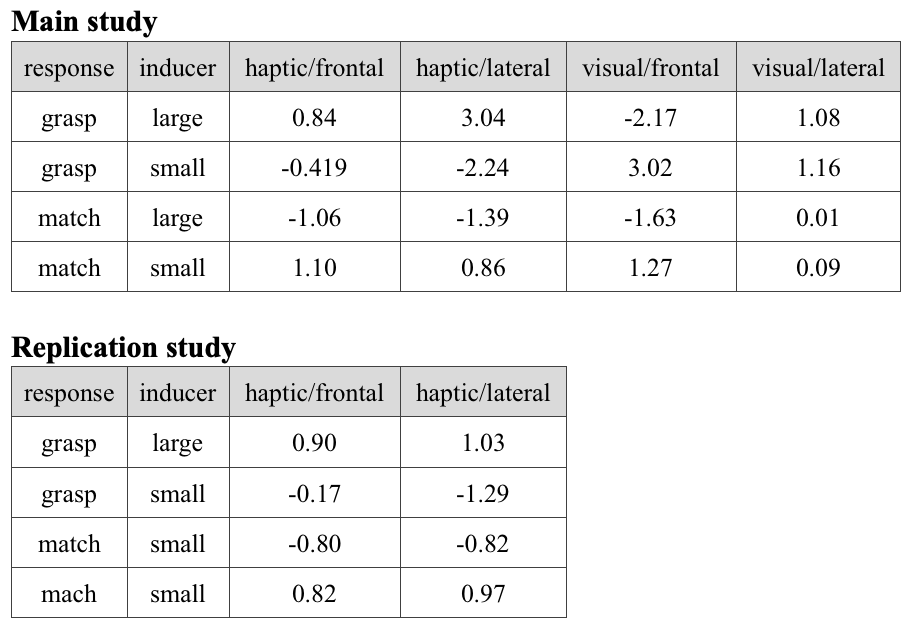

Supplement: S5 Table — Each cell reports the Cohen’s d of the estimated fixed effect extracted from Models (4) (see main text for details). (TIF) [file pone.0301737.s005.tif]
